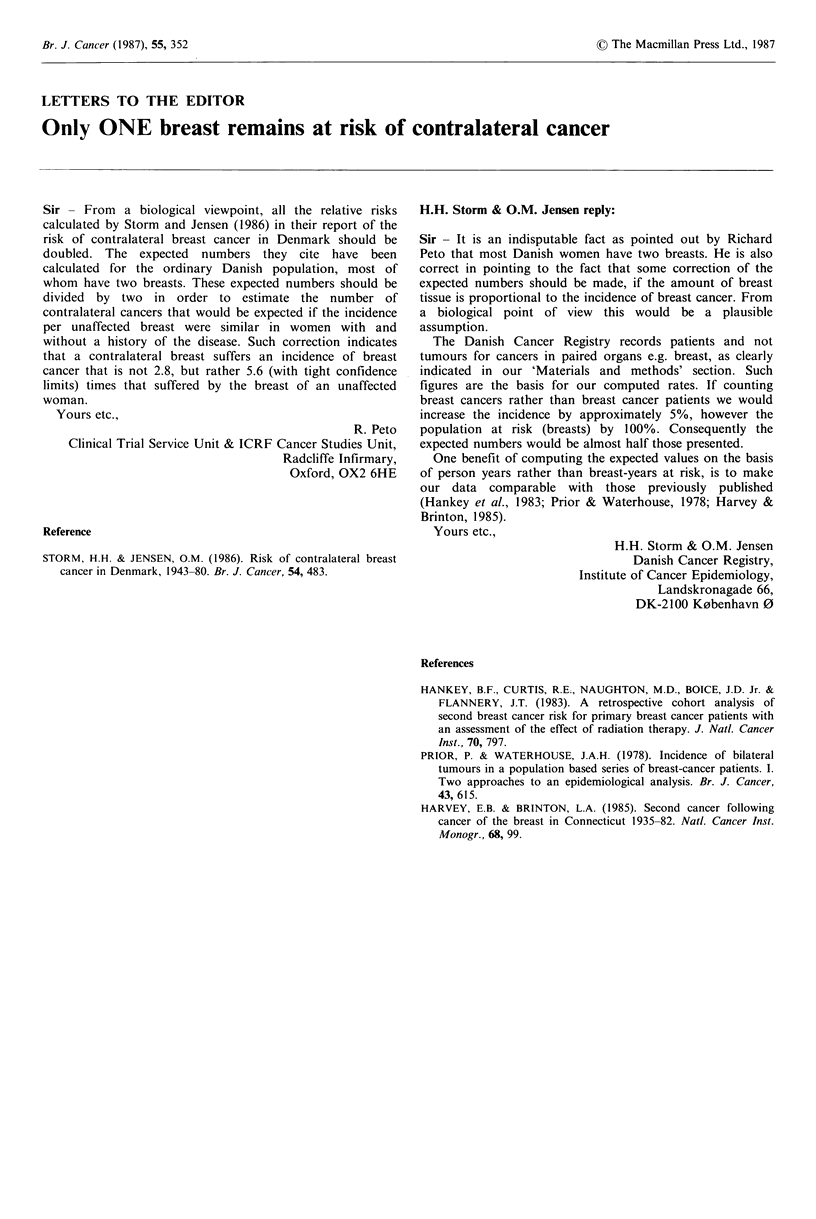# Only one breast remains at risk of contralateral cancer

**Published:** 1987-03

**Authors:** R. Peto


 © The Macmillan Press Ltd., 1987

### LETTERS TO THE EDITOR

### Only ONE breast remains at risk of contralateral cancer

**Sir** – From a biological viewpoint, all the relative risks calculated by Storm and Jensen (1986) in their report of the risk of contralateral breast cancer in Denmark should be doubled. The expected numbers they cite have been calculated for the ordinary Danish population, most of whom have two breasts. These expected numbers should be divided by two in order to estimate the number of contralateral cancers that would be expected if the incidence per unaffected breast were similar in women with and without a history of the disease. Such correction indicates that a contralateral breast suffers an incidence of breast cancer that is not 2.8, but rather 5.6 (with tight confidence limits) times that suffered by the breast of an unaffected woman.

Yours etc.,

R. Peto
Clinical Trial Service Unit & ICRF Cancer Studies Unit,
Radcliffe Infirmary,
Oxford, OX2 6HE

**Reference**

STORM, H.H. & JENSEN, O.M. (1986). Risk of contralateral breast cancer in Denmark, 1943–80. *Br. J. Cancer*, **54**, 483.